# Tele-rehabilitation in Spinal Cord Injury (SCI) in India: An Essential but Uphill Task

**DOI:** 10.7759/cureus.38821

**Published:** 2023-05-10

**Authors:** Anurug Biswas

**Affiliations:** 1 Physical Medicine and Rehabilitation, All India Institute of Medical Sciences, Patna, Patna, IND

**Keywords:** tele-rehabilitation in india, rehabilitation for all, rehabilitation, spinal cord injury, tele-rehabilitation

## Abstract

The number of spinal cord injury (SCI) patients is gradually increasing in India. But due to the unavailability of rehabilitation facilities at the grass root level and most of the patient's financial status, institution-based SCI rehabilitation is still not feasible for many of SCI patients. Tele-rehabilitation can help to rehabilitate the SCI patients to a satisfactory extent where providing hospital-based rehabilitation is not possible. During the COVID-19 pandemic, tele-rehabilitation showed its true potential already. Poverty, lack of education, and lack of technical knowledge of the patients can be a major barrier to its implication. However, with the government’s support, suitable manpower, and will to serve, we can deliver tele-rehabilitation services for SCI patients in the remotest and poorest areas of India.

## Editorial

The burden of spinal cord injury patients and gap in rehabilitation

Based on projections of the latest United Nations data and various International databases, India has now become the most populous country in the world with a population of over 1.42 billion. The incidence of spinal cord injury (SCI) is 9.3-56.1 per million globally depending upon multiple factors [1]. Sadly India still has no database or registry of SCI patients. However, from this number, an assumption regarding the burden of spinal injury in the Indian scenario can be made. Rehabilitation facility for these spinal injury patients is available only in tertiary centers, medical colleges, a few designated rehabilitation centers, and a few private hospitals. Almost all the district hospital, community health centers, and primary health centers lack rehabilitation facilities for SCI patients. According to the National multi-dimensional poverty index about one-fourth of the Indian population is multi-dimensionally poor [2]. It is not always feasible for patients from peripheral and remote parts of India to avail the benefits of hospital-based rehabilitation services. Especially, patients with SCI usually present with multifaceted disability increasing the cost of transport, hospital visit, and stay. The vast majority of these SCI patients and their caregivers are daily wage earners, and hospital visit and stay can jeopardize them financially. Even the SCI patients, who received hospital-based rehabilitation, are not able to revisit due to financial constraints. Moreover, many SCI patients lack community reintegration due to different complications, barriers, and insufficient facilities and arrangements for community rehabilitation.

Scope of tele-rehabilitation in India

Tele-rehabilitation is providing rehabilitation services to patients at their convenient location (without any physical meeting) through modern communication technologies like telephone, video conferencing, social networks, and other platforms. Laptops, computers, smartphone, wearable devices, webcams, etc. are few necessary devices to deliver it properly. Tele-rehabilitation consists of different aspects of rehabilitation like assessment, management, monitoring, education, and prevention. It provides better accessible rehabilitation allowing the communication and transfer of knowledge between patients, doctors, professionals, and caregivers [3]. With the advancement and expansion of technologies, tele-rehabilitation carries immense possibilities in present and future scenario. In a developing country like India, tele-rehabilitation can be an effective and less resource-demanding option [4]. However, just like hospital-based rehabilitation, tele-rehabilitation team should consist not only of the rehabilitation physician (physiatrist) but also the nurse, occupational therapist, physiotherapist, vocational counselor, psychologist, dietician, social worker, etc. During the COVID-19 pandemic, telemedicine services showed their potential and benefits in a populous country like India. However, its incorporation into rehabilitation and its execution is still an uphill task.

How can tele-rehabilitation help the patients with spinal cord injury?

Successful SCI rehabilitation depends on the patient and caregiver’s knowledge and practice. Tele-rehabilitation offers benefit to SCI patients irrespective of the neurological level, stage, and duration of SCI (acute/sub-acute/chronic). Basic things like the positioning of the patient, mattress or cushion type, pressure relieving methods, pressure injury prevention methods, nutritional knowledge, etc. can be easily taught. Scheduled defecation and voiding, assisted defecation, cleaning of the catheter (intermittent catheter), care of catheter, maintaining perineal hygiene, dressing of ulcer, basic physiotherapy and occupational therapy, etc. related advice can be delivered to the patient and his/her caregiver through video conferencing easily. Identification of danger signs of autonomic dysfunction, deep vein thrombosis, respiratory infection, etc. can be done through the use of telemedicine. For previous recipients of hospital-based rehabilitation, new emergence of complication can be dealt up to some extent. Besides communication-related technologies can be used for providing different home and lifestyle modification-related advices. Social reintegration tips, vocational rehabilitation advices, psychologist's counseling, dietician's counseling, etc. are feasible through tele-rehabilitation. In a nutshell, it is possible to assess a SCI patient through tele-rehabilitation along with delivery of prevention and management related advices, education, and counseling [4-5]. The SCI patients are either bed-bound, wheelchair-bound, or use assisted or orthosis-dependent ambulation. To reach the nearest rehabilitation facility, most of them are dependent solely on public or private transport which is an arduous task for a poor patient living in remote areas. For them tele-rehabilitation opens the door for hope and possibilities.

Are we ready for the tele-rehabilitation of spinal cord injury patients?

Though tele-rehabilitation has notable positive aspects in SCI rehabilitation, its implication in India is not at satisfactory level yet. Most of the government and private infrastructure lack proper rehabilitation units for SCI patients, let alone tele-rehabilitation services. Due to financial bindings, most patients from rural areas do not have smartphones or other smart devices, or stable internet connections. Moreover, poor literacy and a lack of minimal awareness of technology can cause hindrances to the successful application of tele-rehabilitation [5]. Government of India is presently making provision of tele-rehabilitation through different tertiary care centers. Telemedicine service is already present in different institutions in India but in most of the cases, it lacks rehabilitation-related aspects due to scarcity of rehabilitation specialists and lack of institutional focus on rehabilitation. A large number of medical colleges in India do not even have a separate, fully functional and properly staffed Physical Medicine and Rehabilitation department to provide rehabilitation-related services to its catchment area. Therefore, setting up proper rehabilitation unit or department in every healthcare facility should be top-most priority before setting up tele-rehabilitation facility. From different studies its cost-effectiveness is already proven. But major barriers to its nation-wide implication are lack of manpower, logistic support, insufficient infrastructure, and other patient-related factors.

Conclusion

As per the author’s experience and present evidence, hospital-based rehabilitation cannot be replaced by tele-rehabilitation in case of SCI. But the major advantage of tele-rehabilitation is superior reach, feasibility, and ease of monitoring. In a country like India, it is the only way to reach each corner. Though the concept of tele-rehabilitation is still in emerging state in India, but with present technology, economic, and literacy growth, it is not an impossible task. With rising numbers of SCI patients, tele-rehabilitation facilities should be developed with priority.
